# An Investigation of the Relationship between Henoch-Schönlein Purpura and Viral Infection in Korea Using the Health Insurance Database

**DOI:** 10.3390/jcm13051290

**Published:** 2024-02-24

**Authors:** So Hyeon Park, Su Min Jo, Sang Won Kim, Jae Min Lee, Hee Sun Baek

**Affiliations:** 1 Department of Medicine, College of Medicine, Yeungnam University, Daegu 42415, Republic of Korea; shyun0120@naver.com (S.H.P.); achiever223@naver.com (S.M.J.); 2 Medical Research Center, College of Medicine, Yeungnam University, Daegu 42415, Republic of Korea; kimsw3767@ynu.ac.kr; 3 Department of Pediatrics, College of Medicine, Yeungnam University, Daegu 42415, Republic of Korea

**Keywords:** Henoch–Schönlein purpura, rotavirus, bocavirus, parainfluenza virus, respiratory syncytial virus, norovirus

## Abstract

(1) Background: This study investigated the epidemiology and viral connections of Henoch–Schönlein purpura (HSP) using information from the Korea Disease Control and Prevention Agency and the Health Insurance Review and Assessment database. (2) Method: Between 2016 and 2019, a total of 25,443 patients with HSP were identified, with 51.3% of patients under the age of 20 years and the highest incidence in March. (3) Results: The autoregressive integrated moving average model and Granger causality test were used to analyze the association between the virus positivity detection rate and HSP incidence. (4) Conclusions: The incidence of HSP was associated with rotavirus, bocavirus, parainfluenza virus, and respiratory syncytial virus in individuals under 20 years of age, whereas adenovirus, respiratory syncytial virus, and norovirus were associated with individuals above that age.

## 1. Introduction

Henoch-Schönlein purpura (HSP) was first described in England by William Heberden, wherein two boys presented with clinical findings suggestive of HSP, including purpuric rash, arthralgia, and abdominal pain [1]. To date, HSP has been studied in children under the age of 18 years but not in adults. HSP is the most common vasculitis in childhood, with an annual incidence of 26.7/100,000, demonstrating that it occurs 2–33-fold more frequently in infants than in adults; recent studies suggest an equal incidence in males and females [2,3]. The diagnosis of HSP is primarily based on clinical manifestations. Recently, several research groups have updated the diagnostic criteria for HSP [4], with palpable non-thrombocytopenic purpura (mandatory criterion) with lower limb predominance in the presence of at least one of the following four features: (1) abdominal pain, (2) histopathology showing typical leukocytoclastic vasculitis with predominant IgA deposition or proliferative glomerulonephritis with predominant IgA deposition, (3) arthritis (acute, any joint) or arthralgia, and (4) renal involvement: hematuria or proteinuria. Although the exact etiology of HSP remains unknown, upper respiratory tract infections are thought to be the cause, and seasonal changes indicate a potential multipathogen connection [5]. In the past, common respiratory tract viruses and group A β-hemolytic streptococcus have been the most common etiologic agents linked to IgA vasculitis. Additionally, during the coronavirus pandemic, there were many case reports showing that the infection caused by SARS-CoV-2 was associated with the occurrence of HSP [6,7,8]. Furthermore, IgA vasculitis has been documented following coronavirus disease (COVID-19) vaccination [9,10,11,12,13]. To determine the correlation between respiratory and gastrointestinal viruses and HSP incidence, we studied the public health data provided by the Korea Disease Control and Prevention Agency (KDCA) and the Health Insurance Review and Assessment (HIRA) Open Access Big Data Platform.

## 2. Methods

### 2.1. Study Population

**Figure 1:**
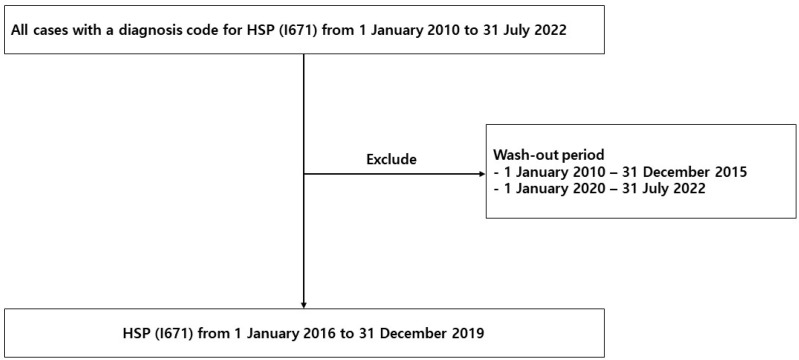
Flowchart for patient selection.

Data regarding HSP were extracted from HIRA, a government-affiliated organization created to build an accurate claims review and quality assessment system for the National Health Insurance, with databases open to all academic investigators. Claims data in the HIRA database include patient diagnosis, treatment, procedures, surgical history, and prescription drugs, which serve as valuable resources for healthcare service research [14,15,16]. We studied the HIRA data of patients with HSP (D690), including HSP cases between 1 January 2016 and 31 December 2019, with all cases having a diagnosis code for HSP (I671) from 1 January 2010 to 31 July 2022. Patients initially diagnosed with HSP from 1 January 2010 to 31 December 2015 and from 1 January 2020 to 31 July 2022 were excluded. The reason for excluding patients before 2015 is that patients diagnosed before 2015 may have been considered as new patients in 2015. The reason for excluding patients in 2020 was that the number of patients may have been underestimated owing to the delay in claiming data (Figure 1).

### 2.2. Surveillance Data of the Virus

We used data reported by the KDCA on viruses that cause acute respiratory infections and gastroenteritis, wherein more than 4000 respiratory and 2000 enteric specimens were collected from 17 local environmental and health institutes and over 100 participating hospitals across Korea during each year of the study period. The causative pathogens were then identified using standardized diagnostic procedures in a central laboratory, in which pathogen prevalence was surveyed weekly and analyzed based on genetic testing of patients with influenza-like illness or acute diarrhea. The positive detection rate (PDR) data were then collected during the study period from 2016 to 2019, calculating the average monthly PDRs of seven respiratory viruses (adenovirus [HAdV], parainfluenza virus [HPIV], respiratory syncytial virus [HRSV], influenza virus [IFV], coronavirus [HCoV], rhinovirus [HRV], and bocavirus [HBoV]) and four acute diarrhea viruses (enteric adenovirus, rotavirus, norovirus, and astrovirus).

### 2.3. Statistical Analyses

For the incidence rate calculations, we used the 2016 Korean population data edited by the Ministry of the Interior and Safety as the denominator, with a total of 51,529,338 people. A model of variations in HSP diagnosis was then constructed using the autoregressive integrated moving average (ARIMA) modeling approach, which assumes that current observations are related to past observations over time. The general multiplicative form of the ARIMA model is denoted as (p, d, and q), where p, d, and q are the order values of the non-seasonal, autoregressive, differencing, and moving average parameters, respectively. Furthermore, the autocorrelation function (ACF) was used to identify the general form of the model. Considering the ACF graphs, different ARIMA models were identified for model selection (Supplementary Figure S1), and the minimum Akaike information criterion model was selected as the best-fit model (Supplementary Table S5). Moreover, the Granger approach was used to investigate the number of current values in the time series that could be described as other values [17,18,19]. Data were analyzed using R software (R Foundation for Statistical Computing, Vienna, Austria, version 4.3.2), and significance was defined as *p* < 0.05 for all analyses.

## 3. Results

### 3.1. Patient Characteristics

During the 4-year period, 25,443 patients were diagnosed with HSP (Table 1). Of these, 13, 063 (51.3%) patients were 0–19 years of age, 3.933 (15.5%) were 20–39 years of age, 4401 (17.3%) were 40–59 years of age, and 4046 (15.9%) were >60 years of age. Regarding sex, there were 11,623 (45.7%) male and 13,820 (54.3%) female patients in this study. 

HSP incidence was the highest in the 0–19-year age group. The HSP incidence rate in the 0–19 years group was 130/100,000 person-years, which was 4.73-fold higher than that in the 20–39 years group.

### 3.2. Trend Analysis of HSP

**Figure 2:**
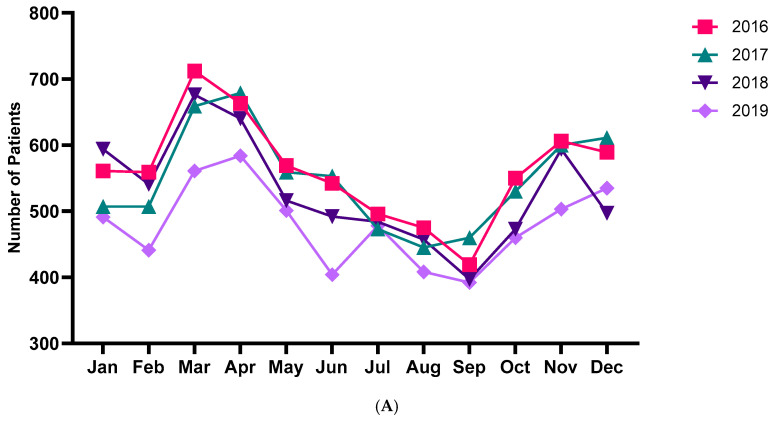

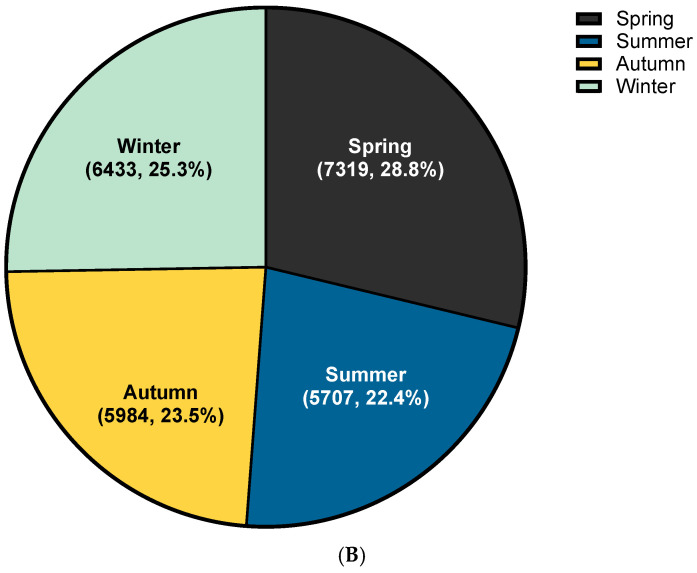
(**A**). Monthly trend analysis of Henoch–Schönlein purpura from 2016 to 2019 according to year. (**B**). Seasonal trend analysis of Henoch–Schönlein purpura incidence.

**Figure 3:**
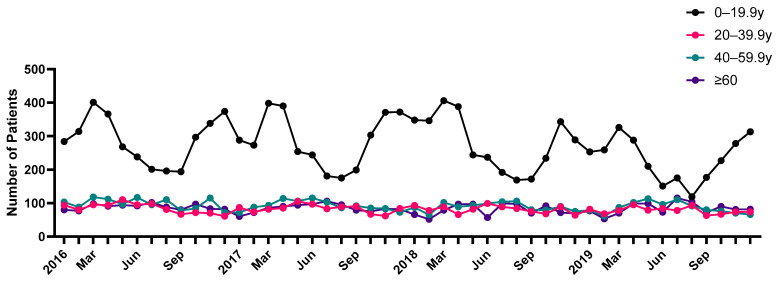
Monthly trend analysis of Henoch–Schönlein purpura according to age group.

Figure 2A reveals the number of patients with HSP per month during the study period from 2016 to 2019. March exhibited the highest incidence rate in the 2016–2019 period (Figure 2A). The cumulative cases per month for 4 years were highest in March and lowest in September. Furthermore, HSP was most frequently diagnosed during spring (28.8%), followed by winter (25.3%), autumn (23.5%), and summer (22.4%) (Figure 2B). Of the 25,443 patients of all ages, 6741 patients had HSP in 2016, 6583 had HSP in 2017, 6361 had HSP in 2018, and 5758 had HSP in 2019 (Tables S1–S3). The average number of cases per month was 212, and the average number of cases per year between 2016 and 2019 was 2544. The seasonal variation in HSP incidence was greater among those aged 19 and younger, while the seasonal variation in incidence was smaller among those aged 20 and older (Figure 3). The incidence tended to increase during spring for all ages.

### 3.3. Clinical Course of HSP

Table 2 shows the clinical course of patients with HSP. Intravenous immune globulin was used for 200 (0.8%) patients. Steroids were used for 9153 (36.0%) patients. Steroid medication duration was as follows: 5 days for 3629 patients, 7 days for 2758 (30.1%) patients, 10 days for 2014 (22.0%) patients, and 14 days for 1430 (15.6%) patients. Of the 25,443 patients with HSP, 7358 (28.9%) were hospitalized, with a hospital visit of 4.30 ± 5.55 days and a duration of hospitalization of 7.87 ± 8.82 days.

### 3.4. PDRs of the Virus

The PDRs of most viruses demonstrated a seasonal variation (Table S4). Specifically, HAdV was the highest from August to November, HPIV was the highest in May, HBoV was the highest from May to June, and HRSV was especially high in winter, with the highest number of HRSV cases in November. Furthermore, the prevalence of HCoV and norovirus was highest from November to January. HRV and enteric HAdV were highest in September, and HMPV, rotavirus, and astrovirus were highest in April, March, and January, respectively.

If any prevalent virus affects HSP diagnosis, the prevalence of the virus may increase prior to the peak of HSP diagnosis. Thus, a Granger causality test was conducted between the viral PDR and HSP diagnostic data collected 1 and 2 months later. The test results are presented in Table 3. Among the eight respiratory and four gastrointestinal viruses, the prevalence of certain viruses increased 1 or 2 months before the HSP incidence increased.

**Figure 4:**
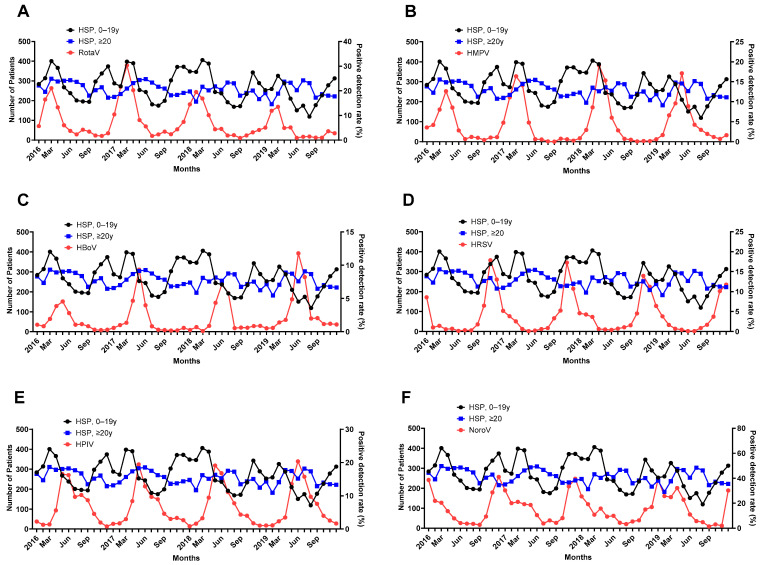
Relationship between the PDR of (**A**) rotavirus, (**B**) HMPV, (**C**) HBoV, (**D**) HRSV, (**E**) HPIV, and (**F**) norovirus incidence of HSP during the study period.

The PDRs for the rotavirus were associated with an increased incidence of HSP after 1 month in the age group > 20 years (*p* = 0.006) and after 2 months in both age groups (*p* = 0.002, *p* = 0.001) (Figure 4A). The PDRs for HMPV were associated with an increased incidence of HSP after 1 month in both groups (*p* = 0.007, *p* = 0.005) and after 2 months in the age group of over 20 (*p* = 0.002) (Figure 4B). The PDRs for HBoV were associated with an increased incidence of HSP after 1 month in both groups (*p* < 0.001, *p* = 0.001) and after 2 months in both age groups (*p* = 0.002, 0.004) (Figure 4C). The PDRs for HRSV were associated with an increased incidence of HSP after 1 month in the age group of over 20s (*p* = 0.002) and after 2 months in the age group of over 20s (*p* = 0.014) (Figure 4D). The PDRs for HPIV were associated with an increased incidence of HSP after 1 month in both groups (*p* ≤0.001, *p* = 0.044) and after 2 months in the age group under 20 (*p* = 0.002) (Figure 4E). The PDRs for norovirus were associated with an increased incidence of HSP after 2 months in the age group of over 20 (*p* = 0.033) (Figure 4F).

## 4. Discussion

HSP can be defined based on the purpura or petechiae as the mandatory criterion and the presence of one or more of the following criteria: acute-onset abdominal pain, arthritis or arthralgia, kidney involvement, hematuria, and proteinuria [20]. HSP is a common form of vasculitis observed in children. 

Globally, the incidence of HSP is 10–20 cases per 100,000 children per year; approximately 90% of cases occur in children between 2 and 10 years of age, with a peak incidence at 4–7 years [21]. Dolezalova et al. reported an annual incidence of 10.2 per 100,000 children under 17 years of age, with a mean age at onset of 7.2 years, and the highest incidence in the 5–9-year age group in the Czech Republic [22]. In a UK study, the estimated incidence was 20.4 per 100,000 and was the highest between the ages of 4 and 6 years (70.3 per 100,000) [23]. Ethnically, it was more prevalent among Asian (24.0 per 100,000) than among Black (6.2 per 100,000) and White (17.8 per 100,000) populations. In a Spanish childhood population study between 1980 and 1990, the overall average annual incidence rate was 10.45 per 100,000 children aged 14 years and younger [24]. In Scotland, the annual incidence ranged from 20.3 to 26.7 per 100,000 people aged < 15 years.

According to the Taiwan National Health Insurance Database study, the annual incidence was 12.9 (11.8–13.4) per 100,000 children under 17 years of age. The occurrence of HSP peaks at the age of 5 to 6 years [25]. The overall hospitalization rate in the United States is 2.4 per 100,000 children under 18 years of age [26]. In Saudi Arabia, HSP is the most common small-vessel vasculitis in children, with an annual incidence of 10–30 per 100,000 [27]. In our study, 51.3% of all patients with HSP were under 20 years of age, with the highest incidence in the 5–9-year age group.

As demonstrated in our study, Dolezalova et al. reported that November and March had two peaks, with the lowest incidence in summer [22]. The seasonal distribution shows that HSP occurs more frequently in winter, whereas the lowest onset occurs in summer [28]. HSP morbidity shows a noticeable seasonal variation parallel to that of some infectious agents. Data on the months of admission in four areas of England and Scotland showed that the incidence of HSP was the lowest in the June to August period. Infection is the most frequent trigger regardless of the clinical phenotype and relapse/recurrence [5].

HSP is a form of vasculitis caused by immune complex deposition [29]. Its etiology and pathogenesis remain unclear; however, a number of factors, mainly infectious agents, drugs, and vaccines, have been considered possible triggers [30].

In a prior investigation by [31] on the prevalence of HSP in Korea, a total of 16,940 patients under the age of 18 were diagnosed between 2013 and 2016. The incidence was highest in the spring (5252, 31.0%) and lowest in the summer (3224, 19.0%). The months with the most and least diagnoses were March (1949, 11.5%) and August (959, 5.7%), respectively. Interestingly, among adolescents, the number of diagnoses was higher in the summer (985, 24.8%) than in the fall (760, 19.1%). The study also found that the epidemic patterns of influenza and rotavirus were temporally and statistically similar to those of HSP across different age groups. The authors speculated that certain viruses, as indicated by seasonality and periodicity, could influence the occurrence of HSP. Our findings further elucidate which viruses affect different age groups.

Using the HIRA database and the data collected by the Korea Centers for Disease Control and Prevention, Hwang et al. studied viral associations in 16,940 Korean patients with HSP aged 18 years or younger between 2013 and 2016 and identified seasonal trends in the incidence of pediatric HSP in Korea and viral etiology according to age [31]. In their study, IFV and rotavirus were significantly associated with the development of HSP in children up to 18 years of age. HRSV, IFV, and norovirus were associated with HSP in early childhood (ages 2–5), IFV and norovirus in middle childhood (ages 6–11), and bocavirus and rotavirus in adolescence (ages 12–18).

In our study, we analyzed patients with HSP of all ages from 2015 to 2019 in Korea using the HIRA database and viral surveillance data from the KDCA. We found that HPIV, HBoV, HMPV, and rotavirus were associated with the occurrence of HSP in children and adolescents under 20 years of age, and HAdV, HPIV, HRSV, HBoV, HMPV, rotavirus, and norovirus were associated with the occurrence of HSP in those over 20 years of age. In our study, 48.7% of all patients with HSP were aged 20 years or older, and we identified different viruses that were more frequently associated with patients aged 20 years or older than with those aged 20 years or younger. HAdV, HRSV, and norovirus appear to be associated with the occurrence of HSP in those aged 20 years and older, whereas they were not associated with those aged 20 years and younger.

Hwang et al. [31] used decomposition additive time series analysis and correlation analysis to compare the incidence of HSP and the prevalence of each virus in patients aged 18 years or younger in three age groups, whereas we used the Granger causality test with an ARIMA model to determine the association between the virus and the development of HSP in all age groups.

Previous studies have demonstrated that respiratory viruses, including PIV, IFV, RSV, and HAdV, are commonly associated with HSP in children [5,31,32]. Chen et al. studied patients with HSP with upper respiratory tract infections and control patients and found that children with HSP frequently have respiratory tract viruses. HPIV plays a significant role in HSP [32]. Weiss et al. analyzed 3132 hospitalizations for HSP using a database of admission records from 40 children’s hospitals in the United States and reported that hospitalizations for HSP increased when hospitalizations for group A β-hemolytic streptococcus, S. aureus, and HPIV increased [33]. HPIV can also contribute to other forms of vasculitis, such as Kawasaki disease [34,35].

Since the identification of severe acute respiratory syndrome coronavirus 2 (SARS-CoV-2), many researchers have conducted related studies. Since 1 December 2019, several HSP cases have been reported after SARS-CoV-2 infection [6,36,37,38,39]. There have been multiple reports of HSP following COVID-19 vaccination [9,40,41,42,43,44]. However, there are reports that the incidence of HSP has decreased significantly during the coronavirus pandemic compared to before [45]. This study demonstrated the role of infection in the pathogenesis of HSP.

Hu et al. [46] also reported that EBV can trigger HSP via cellular and humoral immunity. Several cases of HSP with CMV have suggested that CMV may be a contributing factor [47,48,49]. Shin et al. [50] have reported that hepatitis B surface antigens play a role in HSP development.

In addition to viruses, bacterial infections with Streptococcus, Staphylococcus, and Bartonella can trigger HSP [33,51,52]. Ogura et al. suggested that H. parainfluenza may be a triggering factor, as more antigens and antibodies against H. parainfluenza have been detected in patients with IgA nephropathy [33,53].

The treatment strategies for HSP remain controversial [54]. In 2013, Dudley et al. concluded that treatment with corticosteroids demonstrated no benefit over placebo in reducing the risk of proteinuria 12 months after HSP onset [55]. The SHARE (Single Hub and Access point for paediatric Rheumatology in Europe) initiative in 2019 published consensus treatment recommendations, which included the use of oral prednisolone for mild HSP, with second-line options including azathioprine, mycophenolate mofetil, pulsed methylprednisolone, and similar options, including cyclophosphamide and cyclosporine, for moderate-to-severe disease [56].

Stone et al. conducted a cohort study of 6802 pediatric patients with HSP in the PEDSnet database from January 2009 to February 2020 and reported that 56.8% of patients received no specific treatment within the first year of HSP diagnosis, 36.3% used systemic corticosteroids, and only 7.9% used immunosuppressive medication [57]. In our study, 36% of the patients with HSP were treated with steroids, and the treatment duration varied between 5 days (39.7%), 7 days (30.1%), and 10 days (22%).

Our study had several limitations. First, this was a retrospective study, which provides a lower level of evidence than prospective studies and may have had a selection bias. Second, the data collected from the HIRA database did not include patient clinical records, such as specific clinical courses, outcomes, complications, or the use of drugs not covered by medical insurance. We can only estimate the patient’s clinical progress through the claimed data. Third, the association between the prevalence of certain viruses and the incidence of HSP may not be causal and may have been a chance finding. Since the virus and HSP were discovered at the same time by chance, there are limitations in proving the causal relationship between the virus and HSP. To overcome these limitations, laboratory research is thought to be helpful in revealing the exact cause. Since the Granger causality test is only a statistical analysis method that confirms predictive causality, the basis for claiming causality may be weak. Finally, the data collected by the HIRA only included cases with health insurance; therefore, uninsured cases were excluded.

## 5. Conclusions

In this study, we evaluated the prevalence of common viral pathogens and their correlation with HSP. To the best of our knowledge, this is the largest nationwide analysis of patients with HSP and its association with viral PDRs in Korea. In Korea, an increased incidence of HAdV, HPIV, HRSV, HBoV, HMPV, rotavirus, and norovirus precedes the occurrence of HSP. It is possible that these viruses are the causative agents of HSP. Prospective studies of these viruses and HSP are required to further elucidate these associations.

## Figures and Tables

**Table 1:** Patient characteristics.

Variable			*n* (%)	Annual Incidence *
Total number of patients			25,443	
Age (years)			28.1 ± 25.2	49.2
Age group				
	0–19 years		13,063 (51.3)	130.0
		0~4 years	3893 (15.3)	176.6
		5~9 years	6327 (24.9)	267.9
		10~14 years	1855 (7.3)	78.9
		15~19 years	988 (3.9)	31.6
	20–39 years		3933 (15.5)	27.5
	40–59 years		4401 (17.3)	10.6
	≥60 years		4046 (15.9)	39.9
Sex				
	Male		11,623 (45.7)	45.0
	Female		13,820 (54.3)	53.4
Location				
	Seoul		7936 (23.1)	79.9
	Pusan		2181 (6.4)	62.3
	Incheon		1763 (5.1)	71.0
	Daegu		1922 (5.6)	65.3
	Gwangju		1519 (4.4)	103.4
	Daejeon		1500 (4.4)	99.1
	Ulsan		522 (1.5)	44.5
	Gyeonggi		1742 (22.8)	716.7
	Gangwon		1243 (3.6)	9.8
	Chungbuk		886 (2.6)	57.1
	Chungnam		1424 (4.1)	89.5
	Jeonbuk		1185 (3.5)	56.5
	Jeonnam		784 (2.3)	42.0
	Gyeongbuk		1061 (3.1)	55.7
	Gyeongnam		1959 (5.7)	72.5
	Jeju		629 (1.8)	18.6
	Sejong		3 (0.0)	0.5
Type of insurance				
	Medical insurance		24,533 (96.4)	47.5
	Medical aid		860 (3.4)	1.7
	Free		50 (0.2)	0.1

* All rates are per 100,000 people, directly age-adjusted to the 2016 population.

**Table 2:** Clinical course of Henoch–Schönlein purpura.

Treatment		*n* (%)
IVIG		200 (0.8)
Steroid		9153 (36.0)
Duration of steroid medication		
	5 days	3629 (39.7)
	7 days	2758 (30.1)
	10 days	2014 (22.0)
	14 days	1430 (15.6)
Hospitalization		7358 (28.9)
Hospital visit (days)		4.30 ± 5.55
Duration of hospitalization (days)		7.87 ± 8.82

IVIG, intravenous immune globulin.

**Table 3:** Values of the Granger causality test between the time series of Henoch–Schönlein purpura diagnosis and the time points of positive detection rates of the virus, with <0.05 indicating significance (written in bold).

**A. Diagnostic Data and Virus Data after 1 Month**												
**Age** **Group** **(Years)**	**HAdV**	**HPIV**	**HRSV**	**IFV**	**HCoV**	**HRV**	**HBoV**	**HMPV**	**Rotavirus**	**Norovirus**	**Adenovirus**	**Astrovirus**
0–19.9	0.982	**≤0.001**	0.063	0.798	0.068	0.982	**≤0.001**	**0.007**	0.191	0.889	0.514	0.231
≥20	**0.038**	**0.044**	**0.002**	0.694	0.26	0.828	**0.001**	**0.005**	**0.006**	0.801	0.534	0.526
**B. Diagnostic Data and Virus Data after 2 Months**												
**Age** **Group** **(Years)**	**HAdV**	**HPIV**	**HRSV**	**IFV**	**HCoV**	**HRV**	**HBoV**	**HMPV**	**Rotavirus**	**Norovirus**	**Adenovirus**	**Astrovirus**
0–19.9	0.509	**0.002**	0.271	0.176	0.053	0.3	**0.002**	0.086	**0.002**	0.288	0.833	0.174
≥20	0.064	0.544	**0.014**	0.64	0.221	0.119	**0.004**	**0.002**	**0.001**	**0.033**	0.666	0.872

## Data Availability

The data presented in this study are available upon request from the corresponding author.

## Supplementary Materials

The following are available online at https://www.mdpi.com/article/10.3390/jcm13051290/s1: Table S1: Yearly incidence of Henoch–Schönlein purpura by age group between 2016 and 2019; Table S2: Yearly incidence of Henoch–Schönlein purpura in men between 2016 and 2019; Table S3: Yearly incidence of Henoch–Schönlein purpura in women between 2016 and 2019; Table S4: Positivity rates of virus during the study period; Table S5: Age-based parameters of ARIMA models for patients with Henoch–Schönlein purpura; Figure S1: Residual ACF correlogram and 95% confidence limits for newly diagnosed Henoch–Schönlein purpura. ACF is a statistical technique to determine the degree of correlation between the values in a time series.
