# Supplementary material for: An Investigation of the Relationship between Henoch-Schönlein Purpura and Viral Infection in Korea Using the Health Insurance Database
Source: J Clin Med. 2024 Feb 24;13(5):1290. doi: 10.3390/jcm13051290 (PMC10931917; doi:10.3390/jcm13051290)
Supplement: Supplementary file 1 [file jcm-13-01290-s001.zip › jcm-2858912-supplementary.pdf]

Supplementary Materials

TABLES

Table S1. Yearly incidence of Henoch-Schönlein purpura by age group between 2016 and 2019

Age group	2016	2017	2018	2019	Total
0-4	1080	1029	964	820	3893
5-9	1640	1693	1674	1320	6327
10-14	456	461	508	430	1855
15-19	295	265	222	206	988
20-29	486	527	507	455	1975
30-39	531	478	469	480	1958
40-49	512	480	477	461	1930
50-59	677	631	592	571	2471
60-69	518	462	480	501	1961
≥70	546	557	468	514	2085
Total	6741	6583	6361	5758	25443

Table S2. Yearly incidence of Henoch-Schönlein purpura in men between 2016 and 2019

Age group	2016	2017	2018	2019	Total
0-4	525	524	478	427	1954
5-9	842	873	853	679	3247
10-14	216	238	259	210	923
15-19	131	130	96	85	442
20-29	152	183	170	152	657
30-39	188	173	164	177	702
40-49	218	213	215	182	828
50-59	272	258	225	230	985
60-69	239	221	233	224	917
≥70	250	280	210	228	968
Total	3033	3093	2903	2594	11623

Table S3. Yearly incidence of Henoch-Schönlein purpura in women between 2016 and 2019

Age group	2016	2017	2018	2019	Total
0-4	555	505	486	393	1939
5-9	798	820	821	641	3080
10-14	240	223	249	220	932
15-19	164	135	126	121	546
20-29	334	344	337	303	1318
30-39	343	305	305	303	1256
40-49	294	267	262	279	1102
50-59	405	373	367	341	1486
60-69	279	241	247	277	1044
≥70	296	277	258	286	1117
Total	3708	3490	3458	3164	13820

Table S4. Positivity rates of virus during the study period

Mon PDR(%)	Jan	Feb	Mar	Apr	May	Jun	Jul	Aug	Sep	Oct	Nov	Dec
**HAdV**												
2016	9.18	3.88	3.93	5.54	6.53	6.95	7.16	9.48	9.58	6.50	5.83	3.24
2017	3.72	2.53	2.43	2.74	6.05	4.73	2.96	5.18	4.33	3.78	4.73	2.60
2018	2.98	2.80	4.25	5.38	5.45	6.80	5.82	9.55	13.33	11.30	10.70	7.63
2019	6.68	5.05	4.85	4.43	7.84	10.40	7.52	12.88	10.20	9.44	9.75	8.83
**HPIV**												
2016	2.24	1.30	1.40	5.60	16.55	16.23	9.68	10.25	8.68	4.56	1.95	0.78
2017	1.58	1.70	2.98	8.38	19.43	12.83	9.68	8.95	4.60	3.04	3.33	2.68
2018	0.84	1.60	3.25	9.44	19.10	16.78	11.12	7.75	4.33	4.00	1.75	1.03
2019	1.06	1.10	2.33	3.48	13.56	20.35	15.74	9.70	7.58	3.98	2.55	1.65
**HRSV**												
2016	8.56	1.00	1.43	0.56	0.70	0.15	0.34	0.28	1.78	6.46	17.83	13.02
2017	5.16	3.83	2.53	0.64	0.10	0.23	0.60	0.85	3.30	5.24	17.25	10.73
2018	4.58	4.25	3.65	0.64	0.50	0.40	0.72	1.05	1.55	4.52	13.95	11.20
2019	6.40	3.80	1.65	0.70	0.44	0.10	0.10	0.85	1.68	3.72	10.10	11.73
**IFV**												
2016	15.24	47.55	36.95	26.60	5.00	0.70	0.22	0.28	0.25	0.40	0.80	35.50
2017	26.20	7.70	8.95	12.72	5.03	2.20	1.10	0.55	0.93	0.66	4.05	41.55
2018	58.50	35.63	10.55	3.46	2.80	1.08	0.50	0.30	0.58	1.78	11.15	35.35
2019	28.20	9.13	20.80	34.85	10.52	2.10	0.40	0.35	0.63	3.44	10.08	23.95
**HCoV**												
2016	7.96	3.98	4.95	4.26	4.90	3.08	3.94	4.53	4.68	4.24	7.80	9.82
2017	10.26	6.45	5.15	3.62	2.68	2.35	1.02	1.45	1.55	2.14	5.15	7.20
2018	6.76	9.88	7.73	3.52	0.85	0.48	1.00	3.13	2.60	5.80	10.93	9.83
2019	5.48	3.70	2.83	1.38	0.92	1.68	0.64	0.45	1.05	1.94	3.35	7.55
**HRV**												
2016	8.52	3.78	12.28	15.44	19.55	16.38	19.62	19.60	18.40	24.56	20.68	8.02
2017	9.56	15.40	20.90	18.66	14.78	17.78	21.40	30.63	33.10	24.68	23.88	6.83
2018	2.52	5.95	20.63	28.34	21.53	20.18	21.14	12.70	24.63	22.08	15.70	9.78

2019	8.68	14.33	18.85	16.43	19.82	17.45	23.00	13.45	21.48	22.62	19.65	11.45
						**HBoV**						
2016	1.06	0.83	1.93	3.86	4.55	2.80	1.06	1.15	0.83	0.30	0.25	0.30
2017	0.58	1.00	1.35	4.66	9.33	3.98	0.82	0.30	0.25	0.18	0.20	0.58
2018	0.26	0.70	0.10	0.88	4.35	7.18	5.78	0.55	0.63	0.60	0.85	0.88
2019	0.52	0.58	1.40	1.78	4.88	11.78	8.22	2.00	2.05	1.16	1.20	1.10
						**HMPV**						
2016	3.56	4.20	8.05	12.64	8.53	2.83	0.66	1.20	0.98	0.42	1.05	1.14
2017	4.76	11.10	16.38	14.14	4.80	0.63	0.56	0.10	0.00	0.76	0.60	0.28
2018	0.86	2.95	8.60	19.12	15.33	6.00	2.82	0.73	0.48	0.08	0.18	0.15
2019	0.62	1.68	6.60	9.60	17.10	8.88	4.24	2.98	1.95	1.18	0.70	1.63
						**Group A Rotavirus**						
2016	5.66	16.45	21.08	13.28	6.03	3.73	2.30	4.20	3.43	1.86	1.68	2.76
2017	10.38	21.68	30.33	20.26	8.13	5.53	1.68	2.30	3.45	2.34	4.35	7.38
2018	14.52	19.53	16.88	10.10	4.38	4.55	1.84	1.98	0.85	1.90	3.13	4.10
2019	5.06	11.88	13.48	4.93	5.12	0.90	1.32	1.45	1.00	0.96	3.60	2.75
						**Norovirus**						
2016	38.62	21.85	20.33	13.64	7.80	4.08	3.66	3.40	2.65	9.32	28.58	41.06
2017	30.28	20.00	21.08	19.18	18.58	10.40	3.72	6.20	4.23	8.06	33.40	39.58
2018	25.46	19.20	10.78	15.74	9.30	9.90	4.34	3.20	5.35	6.28	14.95	16.90
2019	39.10	25.70	24.88	32.15	22.74	10.95	5.66	4.80	1.38	2.96	1.93	30.15
						**Enteric Adenovirus**						
2016	2.28	2.58	1.25	2.30	2.28	2.75	2.16	3.43	10.73	6.88	5.68	6.78
2017	2.10	2.23	0.55	2.86	2.90	3.05	2.78	3.30	3.50	5.04	3.43	1.13
2018	2.40	1.85	2.55	3.04	2.85	5.65	4.44	6.45	6.03	3.82	3.33	2.15
2019	0.00	0.85	2.08	2.25	2.00	2.18	1.26	4.08	1.43	1.00	0.80	1.28
						**Astrovirus**						
2016	0.94	1.18	2.48	3.20	3.40	4.30	2.52	2.28	3.43	2.82	2.05	0.86
2017	11.96	1.15	1.73	5.54	3.90	4.08	3.32	2.30	1.78	2.08	0.68	2.58
2018	0.94	1.73	0.00	1.82	3.05	2.50	2.74	5.18	5.63	3.98	2.73	2.33
2019	1.44	3.05	2.70	0.98	1.26	2.20	3.14	1.50	3.23	1.28	4.80	1.50

Table S5. Age-based parameters of ARIMA models for patients with Henoch-Schönlein purpura

Parameters	0-19.9 years	≥20 years	Total
p autoregressive	0	1	0
d difference	0	0	1
q moving average	2	0	0
AIC	522.3	466.1	538.3

AIC, Akaike's Information Criterion.

FIGURES

Figure S1. Residual ACF correlogram and 95% confidence limits for newly diagnosed Henoch-Schönlein purpura. ACF is a statistical technique to determine the degree of correlation between the values in a time series

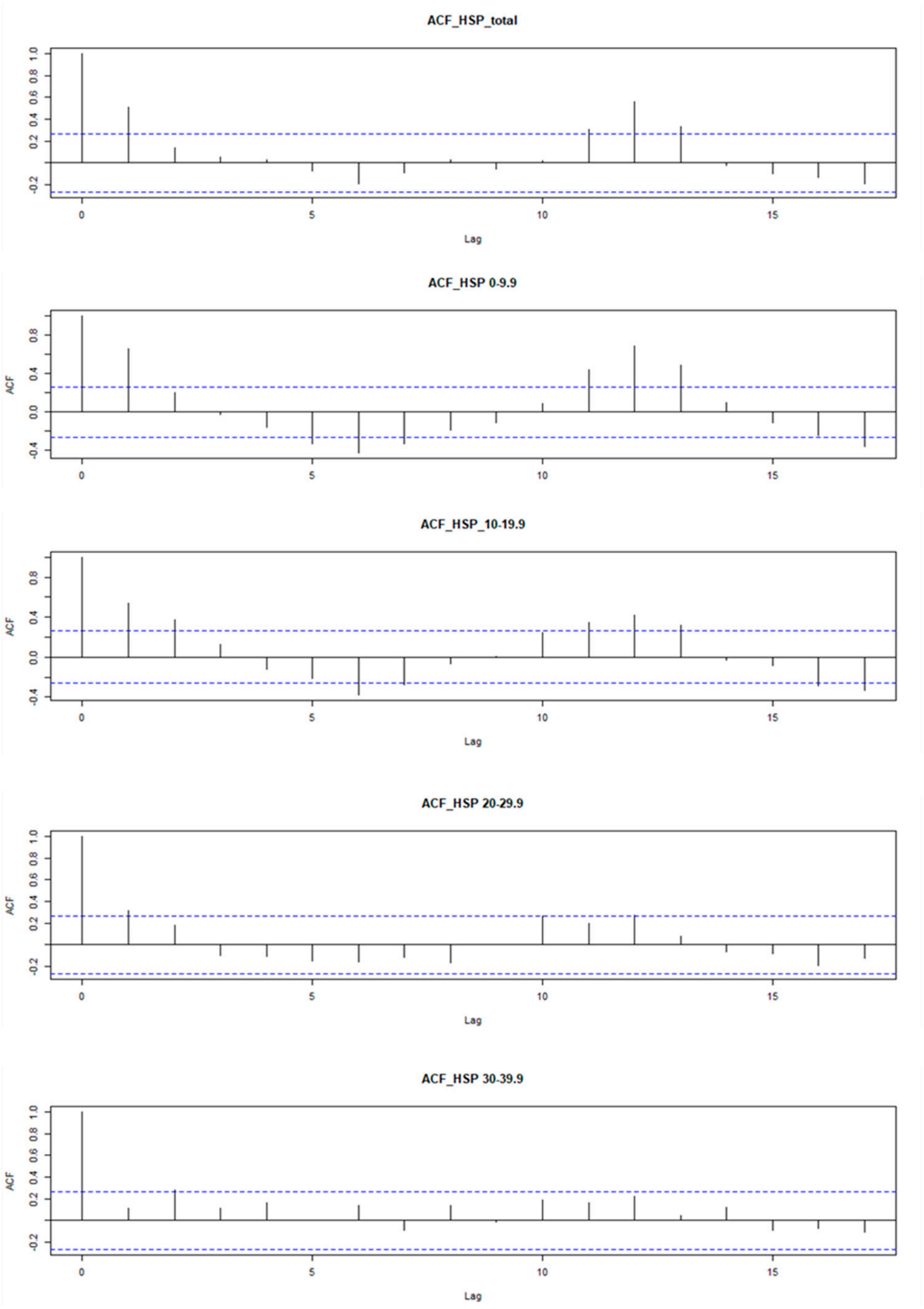

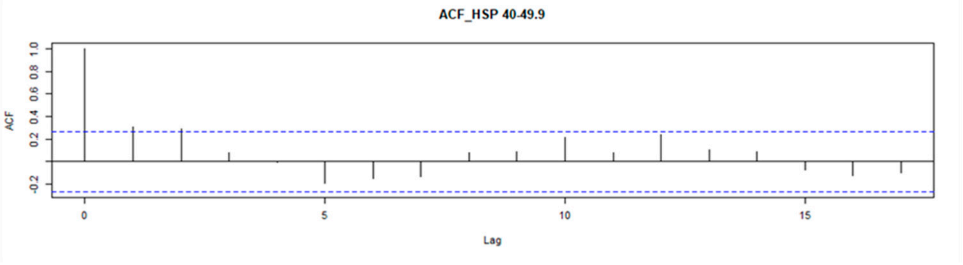
ACF_HSP 40-49.9
ACF
Lag

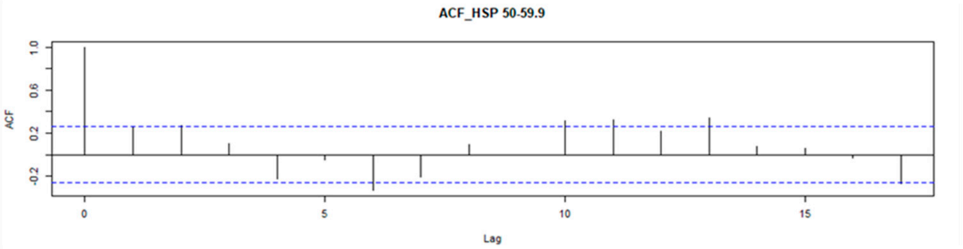
ACF_HSP 50-59.9
ACF
Lag

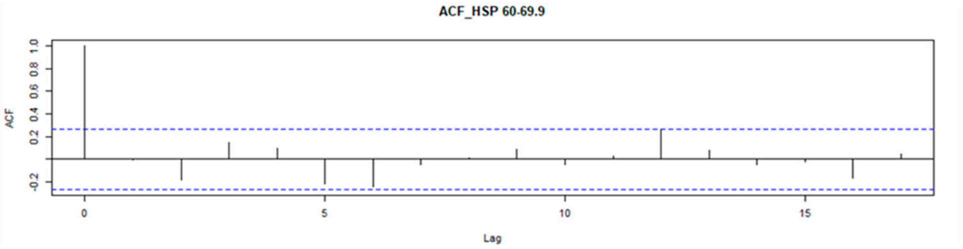
ACF_HSP 60-69.9
ACF
Lag

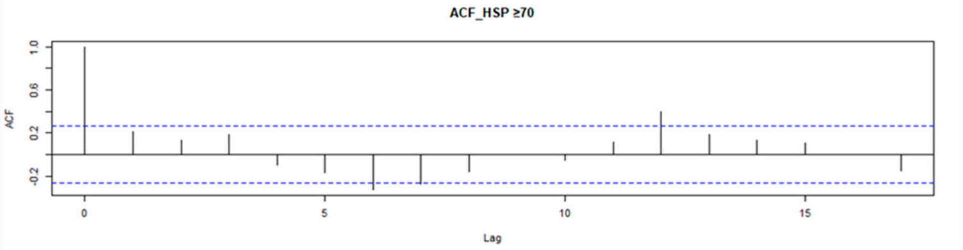
ACF_HSP ≥70
ACF
Lag
